# Arsenic induced Hippo pathway dysregulation linked to hTERT hypo-methylation in breast cancer patients

**DOI:** 10.3389/freae.2026.1853054

**Published:** 2026-06-26

**Authors:** Archana Chaudhary, Manish Kumar, Lalit Mohan, Abhay Kumar, Rishikesh Kumar, Shailesh Kumar, Kumar Pranay, Nandani Kumari, Rizwanul Haque, Md. Margoob Ahmad

**Affiliations:** 1 Department of Pharmacology, Indira Gandhi Institute of Medical Sciences, Patna, Bihar, India; 2 Department of Surgical Oncology, Indira Gandhi Institute of Medical Sciences, Patna, Bihar, India; 3 Department of Microbiology, Indira Gandhi Institute of Medical Sciences, Patna, Bihar, India; 4 Department of Biochemistry, Indira Gandhi Institute of Medical Sciences, Patna, Bihar, India; 5 Department of Biotechnology, Central University of South Bihar, Gaya, Bihar, India

**Keywords:** arsenic, breast cancer (BC), cancer biomarker, human telomerase reverse transcriptase (hTERT), methylation, telomerase

## Abstract

**Introduction:**

A key stage in the development of almost all types of malignancies is the dysregulation of telomeres and telomerase (hTERT). It has been suggested that arsenic-mediated changes in gene methylation status can mute tumor suppressor genes or increase oncogene expression, resulting in long-term alterations in the activity of genes governing cell transformation. Recent study demonstrated that, long-term exposure to an environmentally relevant dose of arsenic affects cellular development by interfering with the Hippo signaling system. The current study’s objective is to ascertain the underlying molecular cause of arsenic induced telomerase alteration and how it is associated with gene methylation.

**Method:**

Twenty-eight female with intermittent breast cancer from arsenic exposed areas were selected, after surgery, a sample of the tumor and the healthy tissue (which serves as a control group) were extracted for further processing. Arsenic concentration was analyzed by atomic absorption spectrometry. Western blotting and RT-PCR were used to analyze the telomerase expression. Additionally, MS-PCR was used to identify the genetic and epigenetic changes, respectively.

**Result:**

According to our research, 65% of female breast cancer patients had extremely high arsenic concentrations with elevated telomerase expression. The cause of this up regulation of telomerase expression is hTERT promoter hypomethylation.

**Discussion:**

The findings also demonstrated a substantial association between breast cancer patients’ upregulation of hTERT and their exposure to arsenic and fine-needle biopsy specimens may provide further predictive information. Higher telomerase expression was associated with a worse outcome.

## Introduction

Despite major improvements in current treatment options, recurrence, metastasis, and drug resistance are still the leading causes of mortality for women with breast cancer (Sung et al., 2021). In recent years, variations in telomerase expression have been linked to various carcinogenesis (Shay, 2016). The expression and rearrangements of telomerase have been found to be altered in several malignancies and are being studied for their potential application as risk factors or early diagnostic markers (Shay, 2016). According to recent studies on a human population exposed to the metal, telomere length is altered by arsenic, which is also linked to chromosomal abnormalities and cancer (Chen et al., 1988). 300 million individuals globally are impacted by drinking groundwater contaminated with arsenic. One of the biggest among them is India, where arsenic poisoning cases have increased in the gangetic plains especially in West Bengal and Bihar. One individual out of every 100 is at high risk of developing the disease due to elevated arsenic exposure (Kumar et al., 2021). Arsenic causes cancers by unidentified mechanisms and is a non mutagenic human carcinogen. Reports suggests that the carcinogenicity of DNA is due to epigenetic changes, such as DNA methylation (Perego et al., 2023; Reichard and Puga, 2010). The activity of genes controlling cell transformation such as hTERT may be altered over time due to long term arsenic exposure, result in methylation of tumor suppressor/oncogene may leads to development of breast cancer (Reichard and Puga, 2010; Chaudhary et al., 2025; Chaudhary et al., 2023). Recent study demonstrated that, long-term exposure to an environmentally relevant dose of arsenic affects cellular development by interfering with the Hippo signaling system. This is accomplished by promoting the nuclear translocation and activation of both the transcription factor TEAD and the primary effector in the Hippo signaling pathway, YAP. The expression of YAP’s target genes, which are involved in the activation and maintenance of pluripotency, increases as a result of this enhanced nuclear accumulation of YAP and TEAD and ultimately leads to tumor development (Mohajan et al., 2021).

According to this article, arsenic causes carcinogenesis, and epigenetic changes result in increased telomerase production, which in turn causes BC. The mechanism of tumor suppressor methylation connected to hTERT dysregulation in the development of carcinogenesis is supported by these data, and it may help to explain the pattern of many and recurrent clinical lesions in arsenic-induced BC.

*In vitro* or *in vivo*, exposure to arsenic has been linked to both hypo- and hyper-methylation at different genomic loci, according to mostly descriptive and sometimes contradicting research (Zhou et al., 2020; Wei et al., 2018). Because of this uncertainty, it has been challenging to determine whether the alterations brought on by arsenic are a direct result of the transformation process or merely a reflection of the changed physiology of rapidly reproducing tumor cells (Chou et al., 2001). In this article, we discuss the evidence that connects changes in DNA methylation to the emergence of arsenic carcinogenesis. The exact pathophysiology of arsenic-induced carcinogenesis is yet understood, however it may involve faulty immune regulation, increased reactive oxidant species, and chromosomal aberrations with unregulated cell growth (Shay, 2016). Therefore, the goal of our research is to clarify the expression of hTERT at the mRNA and protein levels while also assessing its epigenetic and polymorphism changes in arsenic exposed patients with sporadic breast tumor. Our study’s conclusions help us better grasp the hTERT gene’s clinical importance in cases of BC.

## Methods

### Sample collection

The State Cancer Institute, of Indira Gandhi Institute of Medical Sciences (Bihar, India) provided 28 samples of breast tissue that were malignant and an equal number of normal adjacent tissues at the time of surgery. As soon as the tissue was removed, each sample was placed in RNA later and PBS for use in subsequent tests. Using established procedures, the pathologist assessed each sample’s clinical and pathological characteristics, including histological grade and TNM staging.

Each patient has signed a permission document stating that they agree to the sample collection. This study was approved by the ethical committees of Indira Gandhi Institute of Medical Sciences, Sheikhpura Patna, Bihar (Ref N. 1,268/IEC/IGIMS/2023) for its conduct in compliance with the Helsinki Declaration and Good Clinical Practices guidelines. Table 1 displays all of the patients’ demographic, clinical, and pathological data.

**TABLE 1:** A comprehensive information of enrolled female breast cancer patients and their correlation with various clinico-pathological parameters.

S.No	Patient group	Age (Y)	Stage	Ln status	Tumor grading	Geographical location
1	P-1	35	3 B	N1	2	Lakhisaray
2	P-2	40	3 B	N2	2	Buxar
3	P-3	54	3 A	N1	1	Samstipur
4	P-4	41	3 A	N1	2	Purnia
5	P-5	38	3 B	N2	2	Bhagalpur
6	P-6	50	3 B	N1	2	Bhojpur
7	P-10	42	3 A	N1	2	Bhagalpur
8	P-11	27	3 A	N2	2	Patna
9	P-12	34	3 C	N1	2	Buxar
10	P-13	40	3 B	N0	2	Bhojpur
11	P-14	42	3 A	N1	2	Begusarai
12	P-15	36	3 B	N1	2	Bhojpur
13	P-16	42	3 B	N0	1	Siwan
14	P-17	67	3 B	N2	2	Bhojpur
15	P-18	52	3 A	N2	2	Bhagalpur
16	P-19	34	3 A	N1	2	Buxar
17	P-20	55	3 B	N2	2	Bhagalpur
18	P-21	50	3 B	N1	2	Sahabganj
19	P-22	56	3 B	N1	2	Bhagalpur
20	P-23	67	3 B	N1	1	Bhojpur
21	P-22	56	3 B	N1	2	Bhagalpur
22	P-23	67	3 B	N1	1	Bhagalpur
23	P-24	48	3 B	N2	2	Hajipur
24	P-26	52	3 B	N2	2	Gaya
25	P-27	48	3 B	N1	1	Patna
26	P-28	67	3 A	N2	2	Bhagalpur
27	P-29	62	3 A	N1	2	Darbhanga
28	P-30	53	3 B	N1	2	Bhagalpur

### Arsenic estimation in breast tissue

To quantify the quantity of arsenic in breast tissue, 5 ml of HNO_3_ was added to 0.5 g of tissue sample in a 30 ml flask, and the combination was left to react overnight. The next day, every sample was digested for 2 hours at 60 °C on a hotplate. After cooling, 2 ml of HClO_4_ were applied to the samples. A hotplate that was set between 90 °C and 120 °C was used to digest the solution once again for five to 10 minutes, or until the white HClO_4_ fume was expelled. After cooling, 15 ml of distilled water were added to the mixture. The final reading was then obtained by filtering the mixture using Whatman filter paper no. 4, by Graphite Furnace Atomic Absorption Spectrophotometer (GF-AAS). The arsenic content was determined using standard stock solution (1000 μg/L) acquired from PerkinElmer (CAS No. As 7,440–38-2; Lot #: 20-85ASX1; PE #: N9300102).

### Isolation of RNA and DNA

Using the column technique in accordance with the Total RNA Mini and Genomic DNA Mini procedures (Qiagen cata log no-74104 &56,304), total RNA and DNA were extracted from frozen tissue pieces of BC. Following isolation, the Nanodrop 2000 equipment (Thermo Fisher Scientific, Wilmington, DE, United States) was used to spectrophotometrically evaluate the concentration and purity of the RNA &DNA samples. For the reverse transcription procedure, RNA samples with an estimated A 260/280 absorbance value 2 were used. The integrity of the extracted RNA was assessed using 1.5% Agarose gel electrophoresis. Following the manufacturer’s instructions, cDNA was synthesized via reverse transcription in a 20 μL reaction mix using a cDNA synthesis kit (Thermofisher scientific catalog No- 18090050). The final samples were kept for later use at −20 °C.DNA that had been isolated from tissues that were sufficiently pure was kept for later examination.

### mRNA expression by qRT-PCR

The qRT-PCR reaction settings were pre denaturation at 94 °C for 5 min, 94 °C for 45 s, and 60 °C for 60 s, for a total of 40 cycles. The primer sequences used for real-time PCR were hTERT forward (5′-TGGACCAAA GGAAATGTGAACG-3′) and hTERT reverse (5′-TGT​GCA​TAA​ACT​TGA​ACA-3′). GAPDH was selected as an internal reference with primer sequences GAPDH 5′-TGG TAT CGT GGA AGG ACT CAT GAC -3′ and 5′-ATG CCA GTG AGC TTC CCG TTC AGC -3.” LinRegPCR software was utilized to determine the reaction efficiency for each gene. Since all genes had efficiency levels higher than 90%, the Ct counts were converted to fold change using 2^−ΔΔCT^ for further analysis (Schmittgen and Livak, 2008).

### Western blotting

Total protein from both tumor and control tissue was isolated by using RIPA (Radioimmunoprecipitation assay buffer) (Catalog No-89900 from Thermo Fisher Scientific). 20 μg of protein extracts were electrophoreses on a sodium dodecyl sulfate (SDS)-7.5% polyacrylamide gel before being moved to nitrocellulose membrane. The primary antibodies for hTERT (cat # MA5-16034) and the control gene beta actin (cat # 4967 S) were blocked for an hour in fat-free milk and then incubated for the entire night. After three TBST (Tris-buffered saline Tween 20) washes, the membrane was incubated with the appropriate HRP (horseradish peroxidase) labeled secondary antibodies for an hour. Proteins were identified by chemi-luminescence using a clarity western ECL (Enhanced Chemiluminescence) substrate (cat: 170–5,060) (Bio Rad).

### Methylation analysis by MS-PCR

Bisulfite conversion was performed using the EZ DNA Methylation-Gold TM Kit (cat: D5030) in accordance with the manufacturer’s instructions. Using two sets of methylation and unmethylated hTERT primers, the transformed product was amplified. The hTERT gene promoter sequence was obtained from the Eukaryotic promoter database, and primer creation was done using Meth Primer software. The following primer pairs were employed to find methylation in the hTERT promoter region: For the unmethylated detection, sense 5′- TGG​AAA​ATT​GGG​TTT​TAT​TTA​GTT​T-3 and antisense 5′- CAA​CTA​CAA​CTA​CCA​AAC​CAC​C-3′. Antisense 5′- GAC​TAC​GAC​TAC​TTC-3′. The product size was 165 bp for methylation and 164 bp for unmethylation. Initial denaturation at 95 °C for 5 min, 35 cycles of amplification at 95 °C for 30 s, annealing at 53.9 °C (methylation) and 52.7 °C (unmethylation) for 30 s, 72 °C for 30 s, and final extension at 72 °C for 7 min were the conditions used for MS-PCR. The amplified product was seen using the Gel Doc (Bio-Rad Molecular Imaging System) under ultraviolet (UV) light. The images were taken on a 2% agarose gel containing EtBr. There was no difference between the duplicates of the experiments, which were carried out in triplicate.

### Statistical analysis

Graph Pad Prism 8 was used to perform the statistical correlation. The study’s mRNA expression results were presented as mean ± standard error of mean. Non-parametric tests, such as the Wilcoxon signedranked test and the Kruskal Wallis test, were used to correlate the mRNA expression with clinical parameters and assess whether there was a significant difference in Htert/GAPDH mRNA expression. The chi square test was used to correlate protein expression and methylation status with clinical parameters; a p value of less than *<*0.05 is regarded as significant.

## Result

### Clinical profiles of the patients that were enrolled

Breast tissue samples from arsenic affected BC female patients (N = 28) along with adjacent healthy control tissue from same patient (N = 28) were obtained during surgery for the current investigation. Patients were 51.6 ± 10.3 years old on average. Samples were taken from various parts of the breast, such as the nipples, ducts, lobules, and local lymphatic nodes. Both pre and post menopausal women were getting the disease 16 (post menopausal) and 12 (premenopausal) out of 28, suggesting that younger women were also in the high risk to get BC. The Gangetic plains’ riparian zones, which mostly comprise districts like Bhagalpur, Buxar and Bhojpur have a very high disease burden in Bihar (Figures 1a,b). The selected patients’ location, ER, PR, and Her2 neu hormone receptor statuses were examined, and the results were grouped into molecular subtypes. The majority of patients (22/28) had advanced stages (III a and III b), indicating inadequate disease diagnosis (Table 1).

**FIGURE 1:**
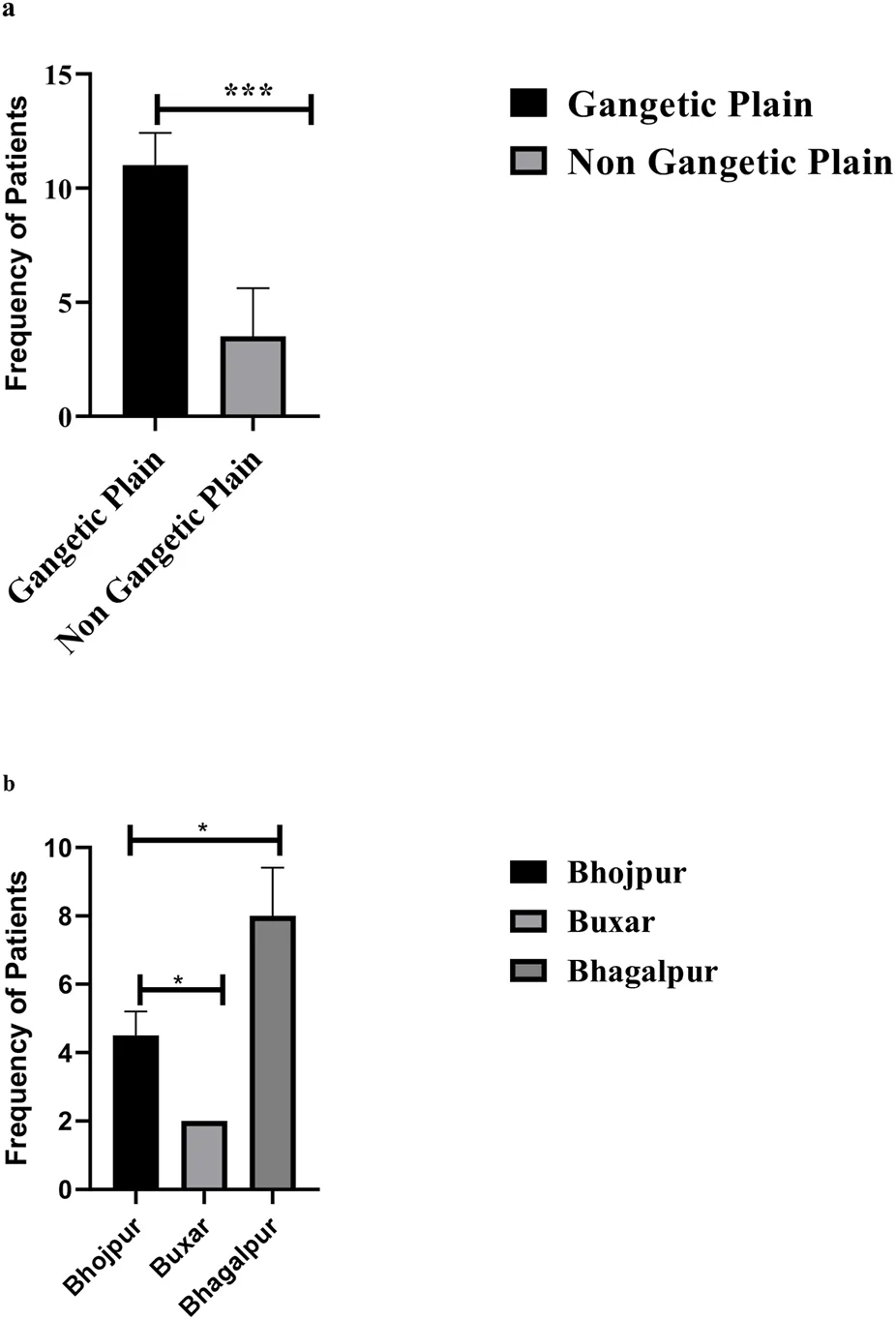
Frequency of breast cancer patients coming from arsenic exposed areas of Bihar: **(a)** Disease burden from various arsenic hit geographical areas of Bihar; **(b)** Graph showing significantly larger number of patients coming from Bhagalpur district of Bihar which has a higher level of ground water arsenic contamination. Statistical significance was determined using a two-tailed unpaired *t*-test (7a, n = 3) or one-way ANOVA followed by Tukey’s post-test (** *p* < 0.01, *** *p* < 0.001). Data are mean ± SEM.

### Arsenic contamination in the breast tissue

In the current investigation, the level of arsenic in the breast tissue of the 28 individuals with breast cancer climbed considerably. The following were the mean amounts of arsenic in breast tissue: Stage I was 15·19 ± 3·85 μg/kg, Stage II was 28·21 ± 8·29 μg/kg, Stage IIIb was 59·17 ± 20·62 μg/kg, and Stage III was 48·80 ± 16·10 μg/kg. This indicates a strong relationship (p < 0.05) between the patient’s location, stage, and exposure to arsenic (Figure 2).

**FIGURE 2:**
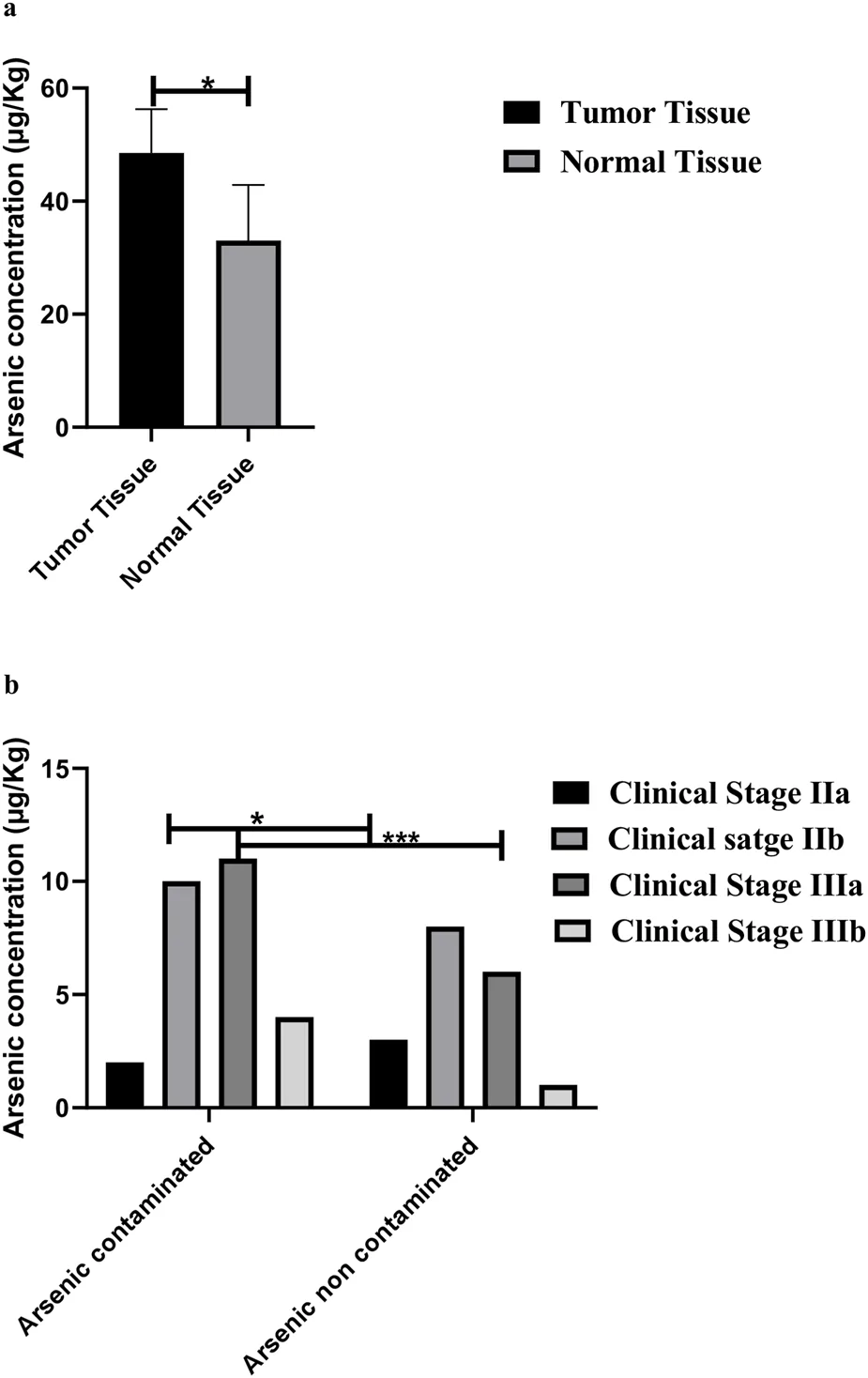
Arsenic accumulation in tumor tissue of arsenic exposed female breast cancer patients: **(a)** Graph showing elevated arsenic concentration in tumor and adjacent tissues of female breast cancer patients coming from arsenic contaminated regions of Bihar; **(b)** The staging of patients increases significantly (p < 0.05) with the increase arsenic contamination. Statistical significance was determined using a two-tailed unpaired *t*-test (n = 3) or one-way ANOVA followed by Tukey’s post-test (7b, n = 5) (** *p* < 0.01, *** *p* < 0.001). Data are mean ± SEM.

### Up regulated telomerase expression in breast tumor

Based on a real-time PCR analysis of mRNA expression, telomerase mRNA is higher in breast tumor tissue than in the nearby control tissue samples. Telomerase expression was normalized using the housekeeping gene GAPDH, and the elevated cases showed a mean fold change 4.2.

In all, 68% (19/28) of the patients showed telomerase over expression while 10% (3/28) showed down regulated telomerase expression and rest 21% (6/28) remains unchanged at the mRNA level, and the higher expression was correlated with clinical data; a significant correlation was found between the patient’s location as maximum of the female patients were coming from arsenic contaminated region of Bihar such as Bhagalpur, Bhojpur and Buxar, which containing higher arsenic, i.e., >300 μg/L age (p = 0.025) (Figure 3; Table 2).

**FIGURE 3:**
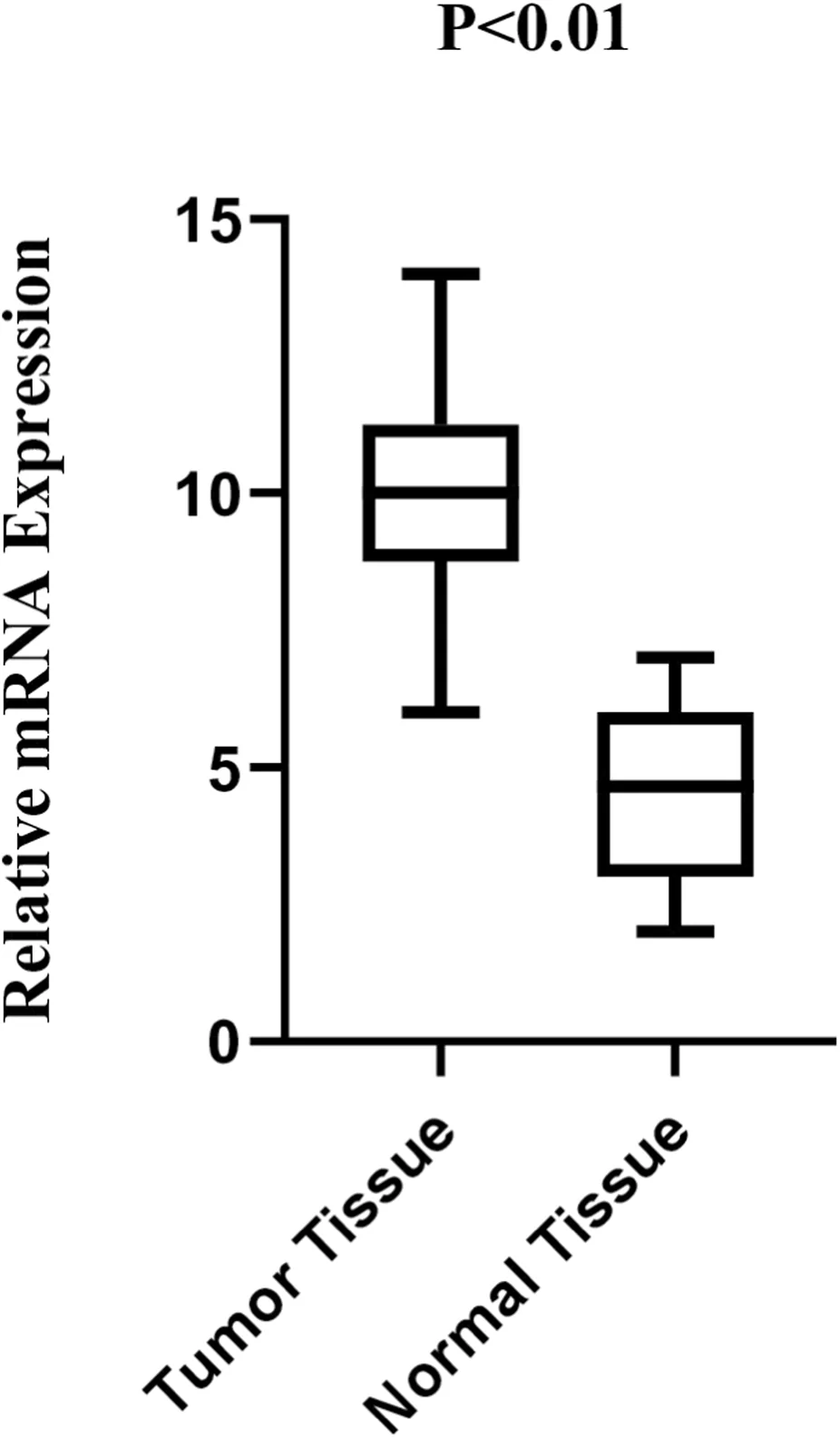
Arsenic induces hTERT expression in arsenic exposed female breast cancer patients: Arsenic metal toxicity significantly unregulated hTERT expression at mRNA level; The q-RT-PCR technique was used to determine the expression of hTERT from adjacent non-tumor and tumor tissue. The relative fold change (2^−ΔΔCT^) of adjacent and tumor tissue (Mean ± SD) is shown. The significance was determined using the two-tailed paired Student's t-test. (** *p* < 0.01, *** *p* < 0.001).

**TABLE 2:** *hTERT* mRNA expression and its correlation with clinicopathological parameters of the patients.

Characteristics	Case	hTERTmRNA expression relative to GAPDH ±SE	P Value
Normal Tumor	28 28	1.01 ± 0.1 2.8 ± 0.12	0.01*
Age ≤50 *>*50	12 (42%) 16 (57%)	3.2 ± 0.27 2.3 ± 0.29	0.02
Menopausal status Premenopausal Postmenopausal	12 (42%) 16 (57%)	3.5 ± 0.18 3.6 ± 0.27	0.8
Estrogen receptor status Negative Positive	8 (28%) 20 (71%)	2.8 ± 0.54 4 ± 0.52	0.173
Progesterone receptor status Negative Positive	9 (32%) 19 (67%)	2.9 ± 0.4 3.0 ± 0.39	0.02
Her2 neu status Negative Positive	12 (42%) 16 (57%)	3.6 ± 0.23 3.3 ± 0.45	0.265
Lymph node status Negative Positive	6 (21%) 22 (78%)	2.1 ± 0.25 2.7 ± 0.34	0.03
Tumor size ≤5 *>*5	5 (17%) 23 (82%)	2.1 ± 0.02 0.32 ± 0.55	0.86
Clinical stage (III + IV) (I + II)	21 (75%) 7 (25%)	2.5 ± 0.43 3.4 ± 0.41	0.495

### Expression of the telomerase protein in breast cancer

The protein-level expression of the telomerase gene was ascertained by comparing and analyzing the findings of western blotting. Majority of the arsenic affected BC patients had over-expressed hTERT in their breast sample. When compared to adjacent tissue, the hTERT protein was significantly up-regulated in breast tumors, and it was enhanced in 57% (16/28) of instances. Our results show a robust correlation between elevated hTERT, estrogen receptor status, Age of the patient and BC prognosis. The mRNA-level expression of the hTERT gene was justified by comparing and analyzing the findings of western blotting. Majority of the high grade breast tumor patients had an up regulated hTERT expression 57% (16/28) both at protein and mRNA level (*p <* 0.007) (Figure 4; Table 3).

**FIGURE 4:**
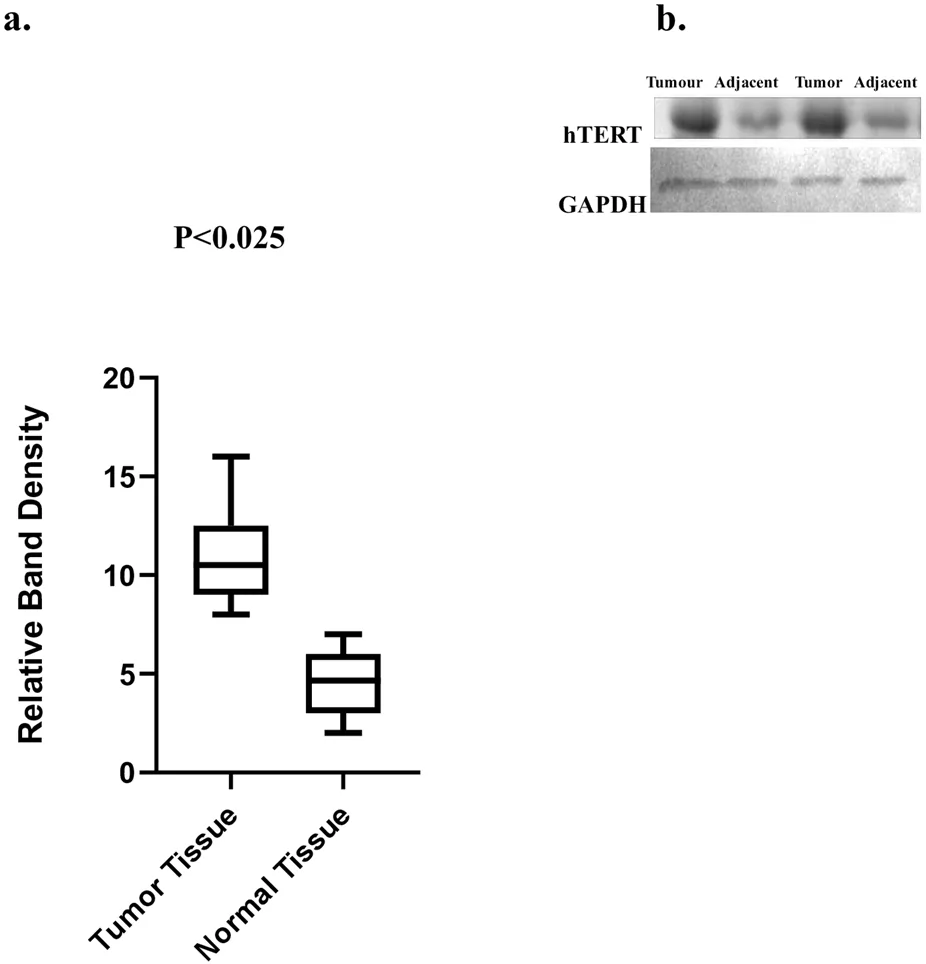
hTERT protein expression analysis through western blot. **(a)** Up regulated hTERT in the tumor tissue as compared to its paired normal breast tissue in arsenic exposed female breast cancer patients of Bihar. **(b)** Comparative analysis of hTERT/Gapdh expression in adjacent and breast tumor tissues. Statistical significance was determined using a two-tailed unpaired *t*-test (7a, n = 3) or one-way ANOVA followed by Tukey’s post-test (n = 5) (** *p* < 0.01, *** *p* < 0.001). Data are mean ± SEM. The original blots/gels are presented in Supplementary Figure S2.

**TABLE 3:** hTERT protein expression and its correlation with clinicopathological parameters of the patients.

Characteristics	Case	High hTERT	Low hTERT	P value
Age ≤50 *>*50	12 (42%) 16 (57%)	10 6	2 10	0.028*
Menopausal status Premenopausal Postmenopausal	12 (42%) 16 (57%)	10 6	2 10	0.02*
Estrogen receptor status Negative Positive	10 (35%) 18 (64%)	2 14	8 4	0.006*
Progesterone receptor status Negative Positive	11 (39%) 17 (60%)	7 8	4 9	0.44
Her2 neu status Negative Positive	12 (42%) 16 (57%)	8 6	4 10	0.13
Lymph node status Negative Positive	5 (17%) 23 (82%)	4 12	1 11	0.342
Tumor size ≤5 *>*5	6 (21%) 22 (78%)	2 14	4 8	0.361
Clinical stage (III + IV) (I + II)	22 (78%) 6 (21%)	16 0	6 6	0.007*

### hTERT promoter methylation and its association with patient clinical factors

Using methylation-specific PCR, the promoter methylation analysis of the hTERT promoter area revealed that 89% (25/28) of the patients had an altered hTERT promoter region with the majority (57%) exhibiting elevated hTERT expression. Furthermore, 10% (3/28) (*p <* 0.0001) instances were regarded as unmodified since their promoter methylation status remained unchanged and similar proportion was 10% (3/28) were methylated (*p <* 0.0001).57% (16/28) of the patients showed up regulation of hTERT expression, indicating a substantial association between the aberrant promoter hypomethylation and up regulation (*p <* 0.002). Additionally, early menopause cases had unmethylated CpGs in the hTERT promoter region, and patients at menopause showed a significant correlation (p = 0.006). Also, the age of the patients at menopause exhibited significant association (p = 0.006) and early menopause cases had unmethylated CpG’s in hTERT promoter region. Hypomethylation of the hTERT promoter region was shown to be significantly (*p <* 0.0001) associated with advanced stage (III and IV) breast cancers in 78% (22/28) of patients (Figure 5; Table 4).

**FIGURE 5:**
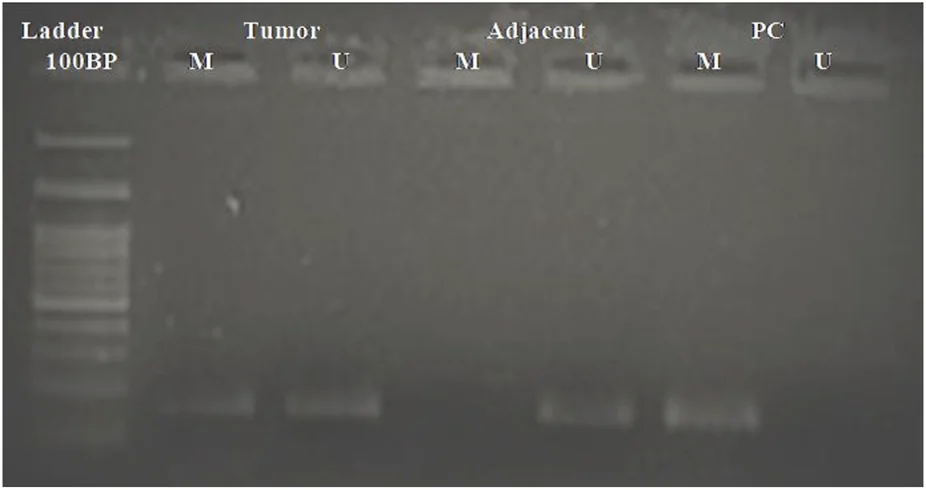
Representative Agarose gel image for MS-PCR. Showing promoter hypo methylation (product size: M = 165) and unmethylation status (product size: U = 164) of hTERT in tumor tissue and adjacent normal breast tissue. NC (Negative Control), PC (Positive Control).

**TABLE 4:** Correlation of *hTERT* promoter methylation status with clinicopathological parameters of the patients.

Characteristics	Case	hTERT methylation	hTERT unmethylation	hTERT unaltered	P value
Age ≤50 *>*50	12 16	2 1	9 13	1 2	0.62
Menopausal status Premenopausal Postmenopausal	12 16	3 0	6 16	3 0	0.006*
Estrogen receptor status Negative Positive	10 18	2 1	7 15	1 2	0.39
Progesterone receptor status Negative Positive	12 16	2 1	8 14	2 1	0.74
Her2 neu status Negative Positive	12 16	0 3	10 12	2 1	0.23
Lymph node status Negative Positive	5 23	1 2	2 20	2 1	0.11
Tumor size ≤5 *>*5	6 22	2 1	2 20	2 1	0.018*
Clinical stage (III + IV) (I + II)	22 6	0 3	22 0	0 3	0.0001**
Characteristics	Case	hTERT hyper methylation	hTERT hypo methylation	hTERT unaltered	​

## Discussion

This study attempt to directly link arsenic to the risk of breast tumor worldwide. People who live in areas where arsenic is present are exposed to the element’s negative effects for an extended length of time, which can cause serious health problems due to modifications in how the body’s systems metabolize it. Toxic levels of arsenic also affect hormones. The xenoestrogenic characteristic of arsenic disrupts several reproductive hormones in females, including oestrogen, progesterone, luteinizing, and oxytocin hormones (Kortenkamp, 2011). The Hippo signaling system in P19 mouse embryonic stem cells was compromised after 32 weeks of chronic exposure to 0.1 μM arsenic (Zhang et al., 2020). Arsenic disrupts the Hippo signaling pathway and increases YAP activation, which raises the expression of YAP’s target genes, ctgf and c-Myc, leads to deregulation of cancer related pathways which is associated with the progression and metastasis of various tumor types (Wei et al., 2018; Zhang et al., 2020). It creates hormonal imbalance, which is the primary risk factor for breast tumor in female patients, in addition to interfering with the function of oestrogen and other associated hormones.

So, in 28 female BC patients, our goal was to assess telomerase gene expression pattern in arsenic affected patient and ascertain its significance to clinical characteristics. The results also show that the majority of BC patients are coming from the Gangetic plains of Bihar have a very high arsenic contamination >300 μg/L like Bhagalpur, Buxar and Bhojpur (Supplementary Figures S1a,b). The study’s key conclusion is that the more advanced the disease, the higher the concentration of arsenic in the breast tissue of women with BC (p < 0.05) as shown in (Figures 2a,b). This new discovery is further supported by the fact that the highest levels of arsenic contamination (68·4 μg/kg) in the breast tissue were found in stages III and higher, which correspond to the latter stages of the illness. Additionally, it was shown that the patients’ age and lymph node status were substantially correlated with the elevated expression of telomerase mRNA (Table 2). A positive axillary lymph node score is regarded as a significant prognostic indicator for breast cancer and is also recognized to play a vital part in the patients’ tumor-free survival (Voordeckers et al., 2004). According to earlier reports, Telomerase activity and tumor size, its stages as well as lymph node status, were found to be significantly correlated by Hoos et al. (Hoos et al., 1998). The better understanding of the various genes mainly affected by arsenic, involved in the breast cancer development, would provide stronger clinical outcomes and patient survival. Research on the connection between arsenic on telomerase activity and genetic alterations linked to breast cancer is still a topic of research. Our current study demonstrated that hTERT mRNA expression is elevated in over 68% of instances of BC (Figure 3). It is interesting to note that telomerase mRNA over expression and ER + status are strongly correlated (Table 2). Additionally, similar results were obtained when protein expression was assessed by western blotting (Figures 4a,b; Table 3). As a result, arsenic exposure may greatly raise the disease’s risk by several times. It is possible to deduce the disease burden from the geographical distribution of BC patients with elevated levels of telomerase in the tumor sample. Similarly this metal toxicity may affect a number of signaling pathways that may be implicated. The signaling pathways linked to BC, including BCL-2, MMP-2, MMP-9, VIM, Snail twist, MT, and MLH, are often elevated and CaspPage 12/26 3, PTEN, E-CAD, and BAX are down regulated in response to recurrent exposure to arsenic. WNT, NRF2, AKT, p53, KRAS, and the majority of TGF-β are also activated (Speer et al., 2023; Davey et al., 2007). Metal exposure has been linked to genetic changes in CHEK2, PALB2, NBN, BRCA1, and other vulnerable genes (Selmin et al., 2019). Telomerase is a good prognostic marker because of the high link between telomerase-positive infiltrating breast carcinomas and lymph vascular invasion, a vital phase in the spread of BC and a survival predictor (Mokbel et al., 2000). Even while abnormal DNA methylation has been discovered in many cancers, we still don’t completely understand how this dysregulation impacts oncogenic gene expression and arsenic exposure. Arsenic activates hTERT and YAP transcription by binding to the hTERT promoter and interacting with the transcription factors of the TEA domain family (Chou et al., 2001; Rivenbark et al., 2013). Moreover, prolonged YAP hyper activation increases hTERT expression, telomerase activity, and telomere length. Hence arsenic induced hTERT promoter hypomethylation and up regulation leads to BC initiation and development, it may be due to deregulation of Hippo pathway which may act as an early biomarker for Breast cancer (Easton et al., 2015; Saintilnord and Fondufe-Mittendorf, 2021). Previous findings demonstrated that YAP increases hTERT expression, which may aid in the development of tumors.

Any epigenetic or polymorphism alterations that could be linked to the aberrant expression of hTERT in breast tumors exposed to arsenic were investigated using bisulfite modified DNA Methylation Specefic-PCR. Patients with increased hTERT expression were shown to have a worse survival rate than those with low hTERT expression. The majority of breast tumor samples (57%) exhibited increased hTERT expression, whereas 89% (25/28) showed altered CpGs in the hTERT promoter region. Among patients with high grade BC in stages III and IV, 78% (22/28) had a strong correlation with the hypomethylated hTERT promoter area (Figure 6) and (Table 4). Inorganic arsenic exposure has been demonstrated in several investigations to alter DNA methylation both random and at particular sites. Researchers found that treatment to sodium arsenite (0.08 – 2 μM) or sodium arsenate (30 – 300 μM) significantly increased methylation at the promoter of tumor suppressor p53 in a dose-dependent manner in human lung adenocarcinoma A549 cells (Mass and Wang, 1997). Additionally, Zhao and colleagues found that a rat liver cell line (RL1215) exposed to arsenic over an extended period of time exhibited global DNA hypomethylation, which was associated with malignant transformation (Zhao et al., 1997). These findings are consistent with increased expression of p16INK4a and RASSF1A due to hypomethylation of these genes in human liver carcinoma cells (Mass and Wang, 1997; Zhao et al., 1997; Delatte et al., 2014). Nevertheless, they contradict the discovery that p16INK4a and hypermethylation were caused by arsenic in lung adenomas (Mass and Wang, 1997). Loss or modification of telomerase functions in response of arsenic, which jeopardizes the regular control of cell division and development (Liu et al., 2015). Cancer cells go through several modifications to modify gene expression programming in order to maintain ongoing development, with DNA methylation changes across the genome playing a significant role (Chandravanshi et al., 2018). It is well known that mRNA levels and DNA methylation state are strongly correlated (Lev Maor et al., 2015; Shukla et al., 2011). When compared to the patients’ clinical parameters, the majority of the unmethylated cases were in advanced clinical stages, and promoter methylation research for the hTERT gene shows hypomethylation in the majority of instances. Additionally, it will be crucial to create a reliable animal model for testing arsenic-epigenetic events at the organism level, along with this to ascertain whether arsenic-mediated epigenetic modifications are heritable. Thus, this research will contribute to the development of treatments and diagnostics that are now missing in the identification and management of disease pathology caused by arsenic.

**FIGURE 6:**
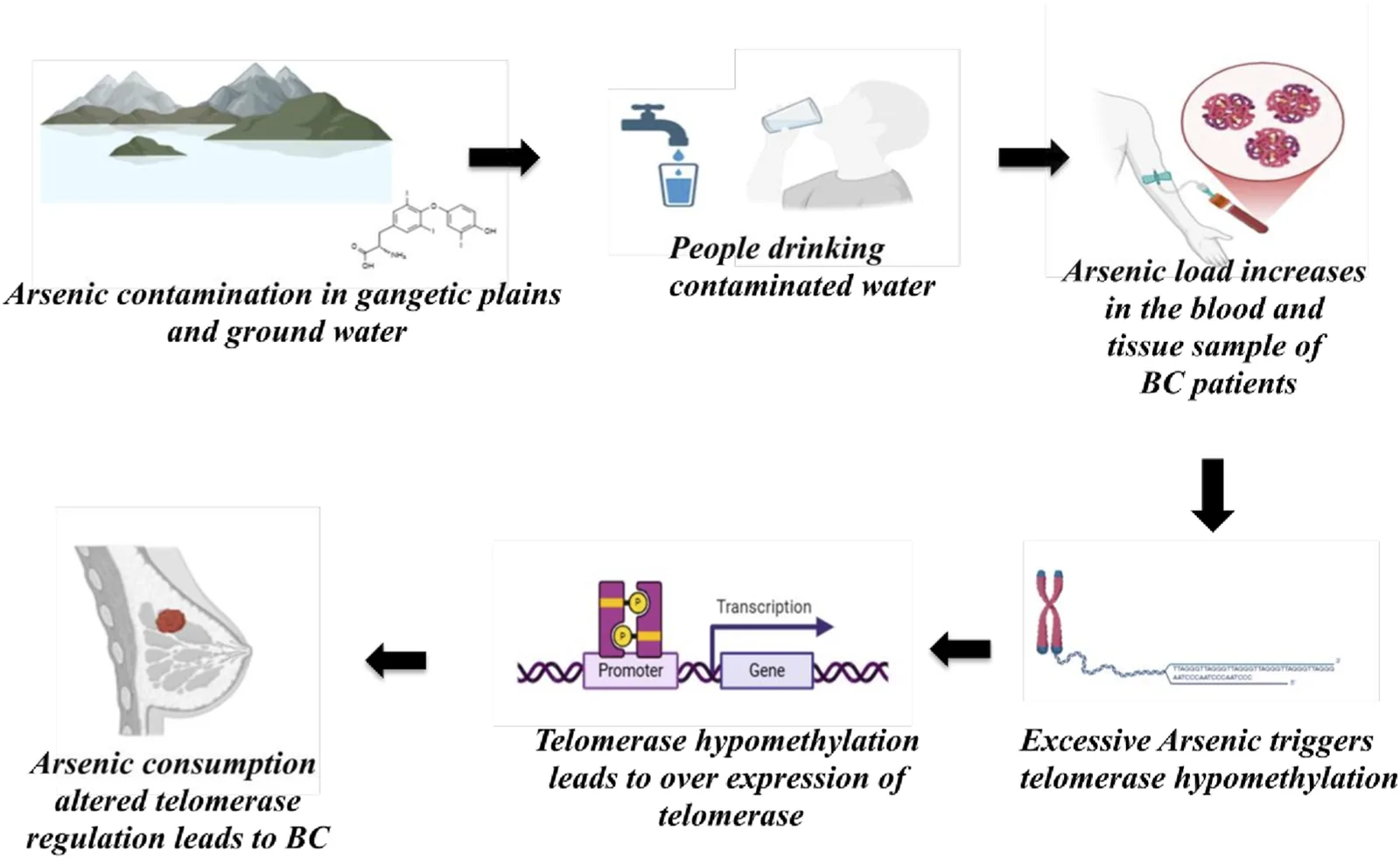
Illustration representing that Inorganic arsenic exposure leads to the hypomethylation of CpGs in the hTERT promoter region which exhibits an over expression of hTERT gene and ultimately causes high grade breast cancer in arsenic exposed breast cancer patients.

## Conclusion

Our research shows that arsenic contaminated patients with breast tumor had greater levels of hTERT expression. According to earlier studies, hTERT has a mysterious role in breast cancer, where it is positively correlated with advanced stages. Our work is the first to reveal that individuals with arsenic induced breast tumor had over expressed the hTERT gene. The majority of these cases had hypomethylated hTERT promoter regions, which were significantly linked to high hTERT gene expression. Our results may serve as a starting point for additional investigation into whether arsenic affect hTERT to contributes BC in an oncogenic or tumor-suppressive manner.

## Data Availability

The datasets presented in this study can be found in online repositories. The names of the repository/repositories and accession number(s) can be found in the article/Supplementary Material.
