# Supplementary material for: Arsenic induced Hippo pathway dysregulation linked to hTERT hypo-methylation in breast cancer patients
Source: Front Epigenet Epigenom. 2026 Jun 26;4:1853054. doi: 10.3389/freae.2026.1853054 (PMC13632481; doi:10.3389/freae.2026.1853054)
Supplement: Supplementary file 1 [file DataSheet1.docx]

**Fig S1.**

**Tumor Tumor Adjacent Adjacent Adjacent Tumor Tumor Tumor**

**hTERT (65KDa)**

**GAPDH (45KDa)**

**Fig S1: Full uncropped Gels/Blots image corresponding to fig 4.**

**Table S2. Primer setup**

| **Types** | **Gene** | **Primer sequence** | **Annealing temperature (°C)** | **Product size (bp)** |
| --- | --- | --- | --- | --- |
|  | **hTERT** | **Methylation**  **F: 5’- TTTTACGGTTATTTAAAGGAACGC-3’**  **R: 5’- TAATTATAAACTCCGACACAACGAT -3’**  **Unmethylation**  **F:5’-TTTATGGTTATTTAAAGGAATGTGG -3’**  **R:5’-TAATTATAAACTCCAACACAACAAT -3’**  **RT-PCR**  **hTERT**  **F 5’ -CCAGGATCAGTAGGTGGAAT-3’**  **R 5’ -GTCTAAAGGTTGTGGGTCTG-3’**  **GAPDH**  **F5’ -TCACCCACACTGTGCCCATCTACGA-3’**  **R 5’-CAGCGGAACCGCTCATTGCCAATGG-3’** | **49**  **56**  **54**  **58** | **165**  **164**  **248**  **452** |

**Table S.2: Table showing primer sequences used for bisulfate based methylation analysis along with primer used in real-time PCR to quantify the expression of hTERT gene.**
